# Changes in hospital efficiency after privatization

**DOI:** 10.1007/s10729-012-9193-z

**Published:** 2012-02-02

**Authors:** Oliver Tiemann, Jonas Schreyögg

**Affiliations:** 1 Hamburg Center for Health Economics, University of Hamburg, Esplanade 36, 20354 Hamburg, Germany; 2 Institute of Health Economics and Health Care Management, Helmholtz Zentrum München, Ingolstädter Landstr. 1, 85764 Neuherberg, Germany

**Keywords:** Hospitals, Privatization, Performance measurement, Data envelopment analysis, Propensity score matching, Germany

## Abstract

We investigated the effects of privatization on hospital efficiency in Germany. To do so, we obtained bootstrapped data envelopment analysis (DEA) efficiency scores in the first stage of our analysis and subsequently employed a difference-in-difference matching approach within a panel regression framework. Our findings show that conversions from public to private for-profit status were associated with an increase in efficiency of between 2.9 and 4.9%. We defined four alternative post-privatization periods and found that the increase in efficiency after a conversion to private for-profit status appeared to be permanent. We also observed an increase in efficiency for the first three years after hospitals were converted to private non-profit status, but our estimations suggest that this effect was rather transitory. Our findings also show that the efficiency gains after a conversion to private for-profit status were achieved through substantial decreases in staffing ratios in all analyzed staff categories with the exception of physicians and administrative staff. It was also striking that the efficiency gains of hospitals converted to for-profit status were significantly lower in the diagnosis-related groups (DRG) era than in the pre-DRG era. Altogether, our results suggest that converting hospitals to private for-profit status may be an effective way to ensure the scarce resources in the hospital sector are used more efficiently.

## Introduction

Rising health expenditure and tight public budgets over the past four decades have led decision makers in many Western industrialized countries to seek ways to improve the performance of health care organizations. Hospitals, in particular, are increasingly being held accountable for their efficiency and financial performance. Having identified inefficiencies and financial risks in public hospitals, national and local governments in a wide range of countries have responded by privatizing these institutions. The chief motivation behind the privatizations that have taken place in these countries during the past twenty years has been the expectation that shifting from public to private ownership would lead to gains in organizational performance, especially in terms of efficiency. Considering the importance of this topic, not least in the political debate, the lack of quantitative empirical studies on the effects of hospital privatization is surprising [1, 2].

The present investigation is the first to examine the effects of privatization on technical efficiency in the hospital market. To do so, we examined a large sample of German hospitals that changed from public to either private non-profit or to private for-profit status. Following an extensive wave of privatizations that began in the mid-1990s, the large hospital market in Germany is now fertile ground for investigating these effects. In Germany, three different types of hospital ownership have co-existed for decades: private for-profit, private non-profit, and public. As their classification implies, both types of private hospitals are owned by private entities, whereas public hospitals are owned mainly by public entities, such as local or regional governments. Between 1995 and 2008, a substantial number of local and regional governments in Germany sold their hospitals to private for-profit and private non-profit owners. The total number of private for-profit hospitals increased by 164, or 44%, which represented a rise in market share from 6 to 18% measured in terms of hospital beds. During the same period, the market share of private non-profit hospitals decreased slightly, from 38 to 36%, because some private non-profit hospitals were also converted to private for-profit ownership [3, 4].

In the present study, we focused on technical efficiency to assess changes in hospital performance following privatization. After using a bootstrapped data envelopment analysis technique to estimate efficiency scores for each hospital in our sample [5, 6], we employed propensity score matching to ensure that the two groups of hospitals in our analysis (i.e. privatized and non-privatized) were comparable in terms of organizational characteristics, environmental characteristics, and patient heterogeneity [7–9]. Subsequently, following an approach suggested by Simar and Wilson [10], we estimated a second-step regression model for truncated longitudinal data, with bootstrapped DEA efficiency scores as dependent variable. Finally, while controlling for the impact of hospital organizational and environmental characteristics, we applied a difference-in-difference specification of the regression model to examine whether privatization improved hospital efficiency.

Section 2 of this paper reviews the relevant theoretical and empirical literature on privatization and its impact on hospital performance. Section 3 presents the setting, data, and methodology used to explore this relationship. Section 4 describes and discusses the results of our analysis, and the fifth, and final, section 5 draws conclusions and suggests topics for future research.

## Previous literature on privatization

Theoretical predictions regarding the performance effects of hospital privatization can be made based on a large body of literature investigating the various effects of different ownership types. There are three fundamental theories that can be drawn upon when comparing public and private hospitals, each of which provides different explanations of a common outcome. According to agency theory and property-rights theory, as well as public choice theory, the rationale for privatization is that the resulting changes in an hospital’s objectives, incentives, and control mechanisms lead to improved performance: Public hospitals acquired by a private for-profit organization are expected to maximize profit through a high degree of technical efficiency, producing services in such a way that marginal cost will equal marginal revenue. In turn, public hospitals acquired by a private non-profit organization are expected to improve technical efficiency by suppressing politically motivated resource allocation and expanding their output at least to the point where total cost equals total revenue [11–18].

Generally speaking, there have been—from an empirical point of view—two categories of studies that deal with ownership and privatization. The first, and by far the most numerous, of these have compared the performance of public and private entities in industries in which both types of ownership coexist [19]. In a meta-review of studies of this nature that focused on the hospital market, Shen et al. [20] concluded that there was little evidence supporting the standard assumption that private for-profit hospitals outperform hospitals with other types of ownership. Herr [21] and Tiemann and Schreyögg [22] found that public hospitals outperform hospitals with other types of ownership in the German hospital market. Because none of these studies investigated changes in ownership type during a specified period—and thus did not examine the effects of privatizing public entities—they will not be discussed in any further detail below.

The second category of studies has focused on privatization and its impact on organizational performance, usually employing a longitudinal design. From a methodological perspective, these studies have taken two different approaches. The first was introduced by Megginson et al. [23], who compared the means and medians of the periods before and after privatization according to defined performance criteria. Few of the studies that have taken this approach, however, have included control groups consisting of non-privatized state-owned organizations. The second approach has involved much larger samples of privatized entities and, defining the privatization event as an intervention, has applied methods proposed in the literature on program evaluation [24, 25]. The majority of these studies have used some kind of difference-in-difference method to analyze the effects of privatization on performance compared to the performance of a control group of non-privatized entities, controlling for time-invariant differences between the groups. As part of our literature review, we were able to identify ten studies from different industries that used such a method to evaluate privatization effects, including four studies on hospitals: Picone et al. [26], Shen [27], Shen [28] and Farsi [29], all of whom examined ownership conversion effects in the US hospital market.

Three of these four studies assessed the quality of care after a change in ownership and found that conversions from private non-profit to private for-profit status had a significantly negative impact [26, 27, 29]. One study [29] found that a change from private for-profit to private non-profit status also had a negative effect on the quality of care. Thus, the negative effect of conversions in both directions may suggest that conversion activity by itself may lead to disrupted organizational routines (e.g., in surgeries) or other organizational problems due to the restructuring process that end up in lower quality of care. Based on this negative effect on the quality of care following conversion activity it seems crucial to control for the quality of care when examining efficiency gains after hospital conversion.

Using profit margins, capacity, staffing ratios, salaries, patient mix, and the amount of unprofitable care as measures, Shen [28] and Picone et al. [26] examined the impact of changes in ownership on hospital performance. Both studies found that hospitals converted from public or private non-profit status to private for-profit status increased their profit margins and reduced staffing ratios. In addition, Shen [28] found that these hospitals reduced cost and increased revenue. She also found that a change from public or private for-profit status to private non-profit status resulted in slight reductions in cost and in nursing staff, but did not lead to increased profit margins.

Although all four studies were pioneering in their approach and focus, they have several important weaknesses. First, Shen [28] and Picone et al. [26] used profit margins as a measure of performance. There is evidence, however, that public hospitals are averse to seeking profit maximization [30]. As a result, it seems reasonable to assume that using financial performance criteria, such as profit margins, may lead to bias in pre-post comparisons of privatized entities and control group designs. For this reason, we believe that productivity, in terms of technical efficiency, is a more suitable measure of changes in performance among hospitals that have undergone a conversion in ownership type. Second, none of the four studies specifically examined the phenomenon of privatization, which would have required a focus solely on changes from public status to private non-profit or private for-profit status. Instead, they examined the effects of conversion from any form of ownership to public, private non-profit, or private for-profit status. Third, Shen [27, 28] was the only author to address the problem of causal interference, using a difference-in-difference method combined with a matching approach to investigate the impact of privatization. Finally, all four studies implicitly assumed that changes in performance are attributable primarily to changes in ownership status. There may, however, be other changes to the market environment, such as the introduction of DRGs, that also interact with the privatization event. By focusing on the impact of privatization on technical efficiency and employing a difference-in-difference matching approach, we attempt to address these shortcomings and contribute to the current understanding of the consequences of hospital privatization.

## Estimation strategy and data

### Data envelopment analysis

In studies on the hospital sector, DEA is the most frequently used approach to measuring efficiency [31, 32]. It is a non-parametric frontier method which uses linear programming to evaluate the relative technical efficiency of an individual hospital based on observed data. Essentially, DEA compares an individual hospital’s observed level of outputs and inputs with the best practice production frontier. This frontier was derived by those hospitals that could maximize output given inputs (output-oriented model) or minimize inputs given output (input-oriented model). DEA applies linear programming to construct a piece-wise linear-segmented efficiency frontier based on observed best practice (for a detailed description of the transformation into a linear programming model, see Charnes et al. [33], Banker et al. [34] and Ozcan [35]).

DEA allows multiple inputs and outputs to be considered simultaneously, which seems particularly well-suited to measuring the efficiency of complex service organizations like hospitals. In contrast to parametric methods such as stochastic frontier analysis (SFA), DEA has the advantage of not requiring that any assumptions be made about the functional form of the production or cost frontier. This reduces the need for a theoretical exposition of the model. SFA is also frequently used to measure efficiency in health care organizations. Because both techniques have their strengths and weaknesses, Coelli et al. [36] propose evaluating their suitability depending on the context of study. As in other investigations that have measured hospital efficiency, our decision to use DEA was driven by data availability [37]: our data set contained a range of input and output variables suitable for DEA, but did not contain input prices, which are an important prerequisite for SFA.

Based on our understanding of the market constraints within the German hospital sector, we assumed variable returns to scale, which may be appropriate when it is impossible to assume that all observed units are operating at an optimal scale [34]. In the health care sector, imperfect competition and budgetary constraints, as well as regulatory constraints on entry, mergers, and exits, can often cause organizations to operate at an inefficient scale size [38]. In light of this theoretical framework, we used the following empirical model in our analysis. In the input-oriented case, we estimated the variable returns to scale model by Banker, Charnes, and Cooper (BCC) as a linear programming model, as follows [34]:

- ϑ_i_, *i* = 1,…,*n* is the hospital’s efficiency, where *n* represents the number of observations (i.e. the number of hospitals).
- ${\vartheta_{\text{i}}} = 1/{\theta_{\text{i}}}$, where *θ*_*i*_ is the inefficiency.

Matrix ${\mathbf{X}} \in {\mathbb{R}^{{{\text{k}} \times {\text{n}}}}}$ refers to *k* observed inputs of *n* compared hospitals, and matrix ${\mathbf{Y}} \in {\mathbb{R}^{{{\text{r}} \times {\text{n}}}}}$ refers to *r* observed outputs of the compared hospitals. Vectors ${{\mathbf{x}}_{\text{i}}} \in {\mathbb{R}^{\text{k}}}$ and ${{\mathbf{y}}_{\text{i}}} \in {\mathbb{R}^{\text{r}}}$ represent the inputs and outputs of unit *i* (i.e. the *i*th columns of matrix **X** and **Y**, respectively). Furthermore, **1** refers to a column vector of ones with a suitable dimension. The DEA efficiency score, which is the reciprocal of the inefficiency *θ*_*i*_, can be obtained by solving the following BCC linear programming model [39]:

1 $$ {\text{s}}.{\text{t}}.\matrix{{*{20}{c}} {\theta * = Min\theta, } \hfill \\ {Y\lambda - {y_0} \geqslant 0,} \hfill \\ { - X\lambda + \theta {x_0} \geqslant 0,} \hfill \\ {{1^{\text{T}}}\lambda = 1,\lambda \geqslant 0.} \hfill \\ } $$

Using DEA efficiency scores for a second-stage regression has been found to result in inconsistent estimates unless these scores are corrected by a bootstrapping procedure. According to Simar and Wilson [5, 6] the statistical properties of the estimated efficiency measures are essential for their interpretations and here a serious problem arises in a two-stage analysis (i.e. DEA followed by some form of regression analysis) from the fact that DEA efficiency estimates are serially correlated. The serial correlation occurs in finite samples due to the fact that perturbations of observations lying on the estimate frontier will in many, and perhaps all, cases cause changes in efficiencies estimated for other observations. In the general multi-output multi-input DEA framework, the bootstrapping procedure seems to offer the only means of inferring the statistical properties of the estimated DEA efficiency scores (i.e. to estimate the bias and variance, and to construct confidence intervals). The basic idea of the bootstrap method is to approximate the sampling distributions of the DEA efficiency estimator by using the empirical distribution of resampled estimates obtained from a resampling simulation. The bootstrapping procedure applied in the present study follows the approach developed by Simar and Wilson [5, 6]. In our case, the bias-corrected scores were derived from 500 bootstrap iterations, which allowed us to improve the statistical efficiency in the second-stage regression (see Section 3.3).

When selecting inputs and outputs for the first stage of our analysis, we followed the example of other studies that have developed DEA frameworks for measuring hospital efficiency [35, 37, 38, 40]. For our purposes, we chose six inputs and two outputs. The first input variable (SUPPLIES) represents the amount spent on supplies per year, including operational expenses, but excluding payroll, capital, and depreciation expenses. Taking into account the relative importance of resource use in terms of labor in the hospital production process, we chose the number of full-time equivalents (FTEs) for the following personnel categories as additional input variables: physicians (PHYS), nursing staff (NURSE), other clinical staff (CLIN), administrative staff (ADMIN), and other nonclinical staff members (NONCLIN).

In our first DEA efficiency model, we used the number of inpatient cases per year in each hospital as the only output variable (INPATIENT). A second model specification served as a sensitivity analysis to test whether the efficiency scores and ranks remained stable when a measure of quality of care was employed as a second output [41]. To do so, we used the average in-hospital mortality rate per year for each hospital to adjust for variations in the quality of care between hospitals. The second output thus represents the number 1 minus the average in-hospital mortality rate per year (1-MORTALITY).

Using output variables like these can be problematic if patient heterogeneity (i.e. case mix) varies systematically across the hospitals in a sample. This is because hospitals with a more complex case mix are likely to receive lower efficiency scores. To address this potential shortcoming, we used patient-level data to condense a comprehensive set of comorbidities to a single numeric score that (a) summarized disease burden and resource use and (b) was sufficiently discriminative for predicting mortality. In order to generate this case-mix index for each hospital, we relied on the comorbidities included in the Elixhauser Comorbidity Index [42]. The Elixhauser Comorbidity Index provides pre-defined weights for each of the comorbidities. We applied these weights to the set of diagnoses of each hospital and determined a case-mix index for each hospital. We rescaled the index to provide values between 0 and 1 used this case-mix index to risk-adjust both of the output variables for the DEA models by multiplying each of the variables INPATIENT and 1-MORTALITY with the case-mix index. Another common approach would have been to use a case-mix index whose weight reflected the relative costliness of DRGs. Carey [43, 44], however, reported that individual-level measures represent a vast improvement over such aggregate case-mix measures when controlling for patient heterogeneity.

A correlation analysis showed that our multiple inputs were positively correlated with our output set. This is an important prerequisite for applying DEA. In the present study, the input variables, in particular, were highly correlated, which suggests that a limited number of inputs might have been sufficient to represent the selected input set in our efficiency assessment. Several authors (e.g., Dyson et al. [45]; Jacobs et al. [38]), however, emphasize that correlation is an aggregate measure of the closeness of two sets of observed data and argue that omitting a highly correlated variable can lead to significant changes in efficiency estimates. As a result, variations in the input levels for individual hospitals may have little impact on the correlation while significantly affecting the measured efficiency. A comprehensive set of inputs and outputs may also increase the chance of identifying the presence of a production technology common to all decision-making units. In addition, Dyson et al. [45] argue that omitting variables to increase discrimination is less effective when using large data samples. Based on these considerations, we chose to use all of our input variables for the DEA model. Table 1 shows the descriptive statistics for in- and outputs of our sample used in the DEA models stratified according to the intervention and control groups.

**Table 1:** Descriptive statistics of in- and outputs for the matched sample

Period	t − 1		t + 1		t + 2		t + 3		t + 4	
	Mean	SD	Mean	SD	Mean	SD	Mean	SD	Mean	SD
For-profit										
Number of hospitals	*n* = 99		*n* = 99		*n* = 90		*n* = 77		*n* = 66	
PHYS	71.46	(73.12)	73.32	(74.91)	71.74	(72.76)	70.47	(75.18)	74.97	(79.41)
NURSE	199.7	(168.4)	188.9	(165.2)	179.3	(153.9)	173.2	(153.8)	177.3	(154.7)
CLIN	127.1	(125.8)	126.7	(126.2)	118.5	(118.9)	114.5	(117.8)	120.5	(121.8)
ADMIN	33.55	(29.81)	33.48	(28.27)	31.85	(26.78)	30.98	(26.50)	30.94	(26.17)
NONCLIN	66.07	(57.01)	56.84	(54.95)	48.97	(47.75)	45.20	(47.25)	43.07	(45.14)
SUPPLIES	14.5	(15.7)	15.1	(15.7)	15.3	(15.3)	14.7	(14.5)	16.1	(15.9)
INPATIENT	13147	(10860)	13386	(11286)	13002	(10649)	12822	(11142)	13537	(11780)
1-MORTALITY	0.974	(0.011)	0.975	(0.010)	0.975	(0.010)	0.976	(0.010)	0.976	(0.009)
Non-profit										
Number of hospitals	*n* = 33		*n* = 33		*n* = 24		*n* = 23		*n* = 22	
PHYS	40.64	(41.08)	42.82	(44.35)	49.04	(50.40)	52.54	(53.04)	55.56	(54.46)
NURSE	132.1	(119.5)	128.6	(122.4)	14.4	(137.62)	151.7	(134.9)	156.4	(133.7)
CLIN	78.3	(84.92)	78.8	(84.97)	91.4	(97.96)	96.3	(99.66)	100.7	(100.8)
ADMIN	22.98	(21.42)	22.57	(23.74)	27.01	(29.52)	27.37	(29.45)	28.67	(29.85)
NONCLIN	60.05	(62.30)	56.37	(62.85)	64.05	(77.76)	59.64	(75.01)	59.73	(77.96)
SUPPLIES	7.8	(6.7)	8.3	(7.3)	9.0	(8.6)	10.1	(9.2)	10.6	(10.1)
INPATIENT	8232	(6668)	8631	(7358)	9773	(8526)	12093	(8612)	10316	(8607)
1-MORTALITY	0.978	(0.009)	0.978	(0.011)	0.977	(0.009)	0.976	(0.010)	0.977	(0.009)
Control										
Number of hospitals	*n* = 128		*n* = 128		*n* = 113		*n* = 99		*n* = 87	
PHYS	65.50	(72.50)	69.62	(76.55)	72.73	(79.68)	71.65	(84.07)	69.66	(80.05)
NURSE	189.0	(172.8)	187.2	(168.4)	191.1	(169.9)	183.8	(165.5)	181.3	(166.8)
CLIN	119.4	(141.5)	120.5	(140.7)	124.2	(143.5)	119.7	(139.4)	121.6	(146.9)
ADMIN	32.70	(33.05)	33.08	(33.47)	33.49	(34.57)	31.57	(32.83)	32.11	(34.43)
NONCLIN	73.81	(77.88)	68.42	(71.34)	68.27	(71.91)	66.26	(71.47)	65.35	(71.25)
SUPPLIES	12.0	(13.4)	13.6	(15.5)	14.5	(16.8)	14.3	(17.6)	14.6	(19.6)
INPATIENT	12435	(10516)	12606	(10587)	13049	(10984)	12630	(10736)	12594	(11278)
1-MORTALITY	0.973	(0.013)	0.973	(0.013)	0.974	(0.011)	0.973	(0.013)	0.973	(0.012)

t − 1 is defined as the year before privatization occurred, while t + 1, t + 2, t + 3 and t + 4 represent the four post-privatization years.

### Propensity score matching

The difference-in-difference estimator has the advantage of eliminating unobserved time-invariant hospital-level effects between privatized and non-privatized hospitals. It does not, however, address the problem of potential baseline imbalance between these two groups, which can be caused when large differences in group characteristics prior to privatization lead to selection bias. For instance, if only smaller hospitals are sold by the state, this may result into smaller hospitals in the intervention group of privatized compared to the control group of non-privatized hospitals. In the case of baseline imbalance, results may be very sensitive to the model specification, and regression analysis may effectively extrapolate outside the support of the data. Methods such as propensity score matching have been shown to avoid unreliable inference in parametric models. For example, if privatized hospitals are systematically smaller than the control group of non-privatized hospitals and we assume that smaller hospitals are generally less efficient than larger hospitals, efficiency gains after privatization will be smaller than they would have been without selection by size, i.e. providing equal hospital sizes in both groups. Whereas the use of matching estimators alone is usually unsatisfactory due to the strong assumption that selection is based only on observables, several authors have proposed that a combination of difference-in-difference and propensity score matching methods significantly increases the quality of non-experimental evaluation results [46, 47].

We thus applied a propensity score matching approach proposed by Rosenbaum [7] and Rubin [8, 9] and extracted a sub-sample of non-privatized hospitals in which the distribution of covariates was similar to that in our sample of privatized hospitals. In the first step, we estimated the conditional probability that any hospital in the two samples had been privatized during the study period given the vector of our defined covariates. The propensity scores were derived by performing a logistic regression. In order to achieve a propensity score model that minimizes the conditional bias, it is important to determine predictors and confounders of the intervention–outcome relationship and to identify predictors of exposure, i.e. we determined variables with significant effect on the probability of being privatized [48]. To determine appropriate predictors of exposure, we tested different variables in the logistic regression models, allowing for interactions between variables (for a detailed description of the variables, see Section 3.3). Subsequently, we calculated the predicted probabilities of belonging to the sample of privatized hospitals. Based on this score, each privatized hospital was matched to one non-privatized hospital in the corresponding baseline year (i.e. one year before the privatization occurred). Because the propensity score was the only pre-intervention measurement, the matching algorithm minimized the absolute differences in propensity score [49]. By using one-to-one matching with replacement i.e. each hospital in the control group could be drawn more than once, the total distance between matched pairs was also minimized; known as optimal matching, this method ensures that conditional bias is reduced to a minimum [7, 9, 50]. The number of pairs in the matched sample was further restricted by using calipers of width equal to 0.2 of the standard deviation. A comparison of different caliper widths found that this width was superior to others at reducing conditional bias in the estimation of intervention effects [51].

We assessed the appropriateness of our propensity score matching by using standardized differences for continuous variables and differences for non-continuous variables as recommended by Austin [48]. Standardized differences represent a good measure of appropriateness for the matching procedure, as they depend neither on the unit of measurement nor on the size of the sample [52]. Furthermore, we applied Hotelling’s T-square statistic to test the reliability of our propensity score matching. Whereas standardized differences and differences rely on the cross-sample difference of each variable included in the propensity score matching model, Hotelling’s T-square test considers whether these differences can be taken as jointly insignificant. In the present study, we divided the sample by propensity score quartiles and conducted the test for each sub-sample [53].

### Difference-in-difference estimates

In our regression analysis, we applied a generalized linear regression model for truncated longitudinal data with DEA efficiency scores as dependent variable. A difference-in-difference specification of the regression model was used to assess whether privatization led to improvements in efficiency while controlling for patient heterogeneity and the impact of hospital organizational and environmental characteristics. Truncated regression models were chosen owing to the truncated distribution of the DEA-based relative efficiency estimates [10]. All of our difference-in-difference regressions were modeled with fixed effects and random effects because the results of the Breusch–Pagan and Hausman tests suggested that the assumption of the random effects specification was also appropriate. The fixed effects estimator generally provides more consistent estimates and may pick up much of the unobserved heterogeneity in the hospital-specific effect. Because of this, the fixed effects estimator was our preferred model. Therefore we only report the fixed effects regression results. Our model was as follows:

2 $$ {\vartheta_{{it}}} = {\beta_0} + {\beta_1}PRI{V_i} + {\beta_2}POS{T_{{it}}} + {\beta_3}PRI{V_i}POS{T_{{it}}} + {\beta_4}{Z_{{it}}} + u_i + {\varepsilon_{{it}}} $$

where *ϑ*_*it*_ is the efficiency of the *i*th hospital at year *t*, *t* = 1,…,13; *PRIV*_*i*_ is a dummy variable for privatization, with *PRIV*_*i*_ being assigned a value of 1 if a hospital was privatized at any time between 1997 and 2007 and a value of 0 if not; *POST*_*t*_ is assigned a value of 1 in the years after privatization and 0 before the year during which the hospital changed its status to private for-profit or private non-profit; and *Z*_*it*_ are observable factors affecting the efficiency of hospital *i* at year *t* (i.e. hospital characteristics, environmental characteristics, and patient heterogeneity) u_i_ is the fixed effect. The random term *ε*_*it*_ is assumed to be gamma distributed.

The variable *PRIV*_*i*_ was included to control for time-invariant differences between privatized hospitals in the intervention group (i.e. hospitals that were converted from public to private for-profit or private non-profit status) and non-privatized hospitals in the control group. The coefficient of interest is the interaction between *PRIV*_*i*_ and *POST*_*t*_, which identifies changes in efficiency after a hospital was privatized relative to efficiency in the comparator group. The difference-in-difference methodology assumes that all other temporal factors affecting hospital efficiency had the same impact on hospitals in the intervention group as they did on hospitals in the control group. We thus assume that any changes over time for which we did not control affected all hospitals in the same way. For sensitivity purposes, and to check the robustness of our estimates, we allowed for four alternative post-privatization periods (i.e. first, second, third, and fourth year). The pre-period was defined as the year before privatization occurred and alternatively year 2 before privatization was used.

An important assumption in our study was that environmental and organizational factors can influence the efficiency of hospitals in addition to a change in ownership status. We assert that considering the impact of such covariates on hospital efficiency provides a better explanation of variation in efficiency and more robust findings on the post-acquisition effects of privatization than the approaches taken in previous studies on this topic, none of which controlled for these effects. The use of control variables is of particular importance when examining the hospital market because there are usually a range of structural and regulatory determinants of efficiency that a hospital itself cannot influence. Table 2 provides a comprehensive outline of our statistical analysis.

**Table 2:** Outline of the statistical analysis

Steps	Implementation	Sample Size
1. Increasing homogeinity of the sample	To ensure the comparability of the hospitals in the sample we	Randomly selected data from 1,878 German acute care hospitals were obtained between 1996 and 2008
	(1) Excluded hospitals providing only psychiatric care, university hospitals, day clinics, hospitals with fewer than 50 beds and more than 2,000 beds	A total of 1,187 hospitals remained in the sample
	(2) Excluded non-privatized non-profit and private for-profit hospitals during our study period	A total of 548 hospitals remained in the sample
	(3) Eliminated hospitals with measurement errors and hospitals that had observations in less than 7 years between 1996–2008	A total of 493 hospitals remained in the sample
2. Identification of intervention and control groups	Seperation of intervention and control groups	Intervention group: 132 privatized hospitals were found, i.e. 99 public hospitals were acquired by a private for-profit hospital and 33 public hospitals were acquired by private non-profit hospitals Control group: 361 public hospitals
3. Data Envelopment Analysis (DEA)	Application of an input-oriented variable returns to scale model by Banker, Charnes, and Cooper (BCC) for all privatized and non-privatized hospitals. DEA was performed per year.	
4. Bootstrapping	Derive bias-corrected DEA efficiency scores from 500 bootstrap iterations	
5. Proponsity Score Matching (PSM)	(1) Estimate probability for being privatized based on our defined covariates by means of logistic regression	
	(2) Match privatized hospitals to non-privatized public hospitals based on predicted means by using a one-to-one matching with replacement; we used an optimal matching algorithm i.e. we minimized the total distance of matched pairs	Number of hospitals in control group after matching: 128 in first year after matching, 113 in second year after matching, 99 in third year after matching, 86 in fourth year after matching
6. PSM diagnostics	Assessing appropriateness and reliability of the PSM results by using	
	(1) standardized differences and	
	(2) Hotelling’s T-square statistic based on quartiles	
7. Regression analysis	Use of a generalized linear regression model for truncated longitudinal data with DEA efficiency scores as dependent variable. Difference-in-difference interaction was used to examine effects of privatization on efficiency compared to control group of non-privatized hospitals controlling for patient heterogeneity and the impact of hospital organizational and environmental characteristics	
8. Sensitivity analysis	(1) Re-estimation of the DEA model by employing 1-MORTALITY as a second output	
	(2) Estimated multiway clustered regression including time and firm fixed effects	
	(3) Allowed pre-privatization period of two years instead of one year	
	(4) Re-estimation of the DEA model without those inputs having a strong correlation	
	(5) Reduction of of the multi-step procedure to a substantially reduced two-step procedure	

In the second stage of our analysis, heterogeneity in hospital characteristics was covered by several variables. The first of these was the number of licensed and staffed beds (BEDS), an approach taken in previous studies to control for hospital size [54–56]. In the context of hospital planning in Germany, the number of beds per hospital can be seen, at least over the medium term, as an exogenous factor that is not under the direct control of hospital management. To account for higher resource consumption due to differences in teaching activities, we included a variable (TEACHING) for the training activities of non-medical staff. These activities are represented by the ratio of trainee positions and the sum of all non-medical personnel. Another important point to consider is that hospitals in Germany are allowed to lease hospital beds to self-employed private-practice physicians, although the vast majority of hospital physicians in Germany are employees. The self-employed physicians use the hospital facilities to provide inpatient care. The DEA efficiency scores calculated in the first stage of our analysis were higher for hospitals that had leased beds because the cases referred to these physicians had been counted as hospital output (i.e. inpatient cases), whereas the corresponding resource use in terms of physicians had not been considered on the input side. To control for this, we defined the proportion of all hospital beds that had been leased (LEASED BEDS) as a variable in our regression models.

The set of explanatory variables representing the different environmental characteristics were as follows: The Hirschman-Herfindahl index (HHI) was applied, which measures competitive pressure in a hospital’s market and is a standard economic measure of market concentration. The market area was defined as the county in which a hospital was located, which is a frequently used definition in hospital studies [57–61]. Although there has been some controversy about the most appropriate way to define a hospital’s market area, Garnick et al. [62] reported that for the purpose of measuring competitive activity it makes little difference whether a hospital’s market is defined as a county or a radius. The HHI is obtained by squaring a hospital’s regional market share (reflected by the distribution of treated cases) and subsequently summing the market shares of admissions for all of the hospitals in a given county. The higher the HHI, the more concentrated the regional market. We used HHI to measure the effects from the changes over time in a hospital’s competitive environment. This specification allowed us to differentiate between the effects of privatization and the effects of changes in market structure resulting from health care reforms. In recent years, the most significant reform in the German hospital market was the introduction of a new system of reimbursement based on DRGs. The chief motivation behind this fundamental overhaul of the old reimbursement system, which was based on per diem charges, was to create financial incentives that would increase hospital efficiency [63]. We thus defined a dummy variable (DRG), which was assigned a value of 1 in the DRG era (i.e. 2003 through 2007).

These variables for hospital and environmental characteristics were also used in the propensity score model, as were the case-mix index (CMXI) derived from the Elixhauser Comorbidity Index [42] and the pre-privatization efficiency (EFFICIENCY) of privatized and non-privatized hospitals. We used this last variable to minimize potential bias because public entities may prefer to sell hospitals characterized by lower levels of efficiency while keeping those that are performing well. Table 3 shows the descriptive statistics for the variables used in the second stage analysis stratified according to the intervention and control groups.

**Table 3:** Descriptive statistics of variables used for the second stage analysis for the matched sample

Period	t − 1		t + 1		t + 2		t + 3		t + 4	
	Mean	SD	Mean	SD	Mean	SD	Mean	SD	Mean	SD
For-profit										
Number of hospitals	*n* = 99		*n* = 99		*n* = 90		*n* = 77		*n* = 66	
BEDS	344	(268)	344	(273)	332	(261)	327	(262)	342	(272)
LEASED BEDS	0.037	(0.074)	0.032	(0.073)	0.028	(0.050)	0.026	(0.047)	0.024	(0.047)
TEACHING	0.146	(0.154)	0.171	(0.177)	0.162	(0.191)	0.177	(0.202)	0.129	(0.147)
CMXI	0.161	(0.063)	0.155	(0.061)	0.160	(0.056)	0.154	(0.052)	0.147	(0.048)
EFFICIENCY	0.761	(0.122)	0.757	(0.121)	0.772	(0.123)	0.774	(0.114)	0.772	(0.117)
HHI	0.399	(0.294)	0.393	(0.284)	0.413	(0.291)	0.450	(0.288)	0.443	(0.267)
Non-profit										
Number of hospitals	*n* = 33		*n* = 33		*n* = 24		*n* = 23		*n* = 22	
BEDS	237	(179)	232	(189)	264	(220)	267	(214)	274	(216)
LEASED BEDS	0.060	(0.067)	0.055	(0.064)	0.037	(0.047)	0.039	(0.047)	0.055	(0.097)
TEACHING	0.151	(0.156)	0.165	(0.159)	0.141	(0.151)	0.182	(0.125)	0.099	(0.138)
CMXI	0.141	(0.042)	0.146	(0.044)	0.142	(0.043)	0.128	(0.034)	0.132	(0.037)
EFFICIENCY	0.730	(0.144)	0.757	(0.131)	0.743	(0.129)	0.738	(0.140)	0.716	(0.157)
HHI	0.404	(0.210)	0.439	(0.253)	0.463	(0.274)	0.469	(0.278)	0.475	(0.285)
Control										
Number of hospitals	*n* = 128		*n* = 128		*n* = 113		*n* = 99		*n* = 87	
BEDS	332	(257)	329	(256)	336	(257)	321	(248)	319	(250)
LEASED BEDS	0.048	(0.078)	0.045	(0.078)	0.047	(0.083)	0.043	(0.078)	0.051	(0.094)
TEACHING	0.148	(0.153)	0.146	(0.158)	0.148	(0.158)	0.142	(0.154)	0.146	(0.160)
CMXI	0.162	(0.072)	0.162	(0.070)	0.160	(0.067)	0.154	(0.050)	0.157	(0.061)
EFFICIENCY	0.765	(0.127)	0.746	(0.121)	0.750	(0.129)	0.747	(0.133)	0.747	(0.119)
HHI	0.390	(0.231)	0.394	(0.241)	0.395	(0.242)	0.390	(0.249)	0.398	(0.247)

t − 1 is defined as the year before privatization occurred, while t + 1, t + 2, t + 3 and t + 4 represent the four post-privatization years.

### Sample

The data for our study were derived from the annual hospital reports collected and administered by the Research Data Centre of the Statistical Offices of the Länder. This rich dataset covers all public, private for-profit, and private non-profit hospitals in Germany and contains hospital-level information on cost and hospital infrastructure, as well as patient-level information on age, diagnoses, and procedures performed. Our study is based on data from the years 1996 through 2008, and the unit of analysis was the hospital. Due to data privacy issues, we were able to obtain randomly selected data for only two-thirds of German acute care hospitals (*n* = 1,878). To ensure the comparability of the hospitals in the sample, the following institutions were excluded from further analysis: hospitals providing only psychiatric care; university hospitals; day clinics; hospitals with fewer than 50 beds or more than 2000 beds; and private non-profit and private for-profit hospitals that had been privatized before the study period. In addition, manual plausibility checks were conducted to identify any measurement errors. Ultimately, a total of 548 public hospitals remained in our sample, including 132 that were privatized between 1997 and 2007. Of these 132 hospitals, 99 were acquired by a private for-profit organization and 33 were acquired by a private non-profit organization [64]. Table 4 shows the number of hospital privatizations per year.

**Table 4:** Number of hospital privatizations per year

Period	Number of privatizations		
	Total	Non-profit	For-profit
1997	15	5	10
1998	13	8	5
1999	7	0	7
2000	4	2	2
2001	15	0	15
2002	13	2	11
2003	14	3	11
2004	11	2	9
2005	13	1	12
2006	16	2	14
2007	11	8	3
Total	132	33	99

## Findings and discussion

Table 5 shows the degree of imbalance before and after propensity score matching for our subset of covariates (interactions are not shown).

**Table 5:** Balance in measured covariates before and after matching

Group	Unmatched sample					Matched sample				
	Control (*n* = 361)		Intervention (*n* = 132)		*d*_*i*_	Control (*n* = 128)		Intervention (*n* = 132)		*d*_*i*_
	Mean	SD	Mean	SD		Mean	SD	Mean	SD	
Variable name										
BEDS	351	(259)	317	(252)	12.89%	332	(257)	317	(252)	5.68%
LEASED BEDS	0.069	(0.091)	0.043	(0.073)	2.60%	0.048	(0.078)	0.043	(0.073)	0.50%
TEACHING	0.169	(0.172)	0.147	(0.154)	2.20%	0.148	(0.153)	0.147	(0.154)	0.10%
CMXI	0.157	(0.058)	0.156	(0.059)	0.10%	0.162	(0.072)	0.156	(0.059)	0.60%
EFFICIENCY	0.772	(0.111)	0.753	(0.128)	1.90%	0.765	(0.127)	0.753	(0.128)	1.20%
HHI	0.401	(0.242)	0.400	(0.275)	0.10%	0.390	(0.231)	0.400	(0.275)	1.00%

The differences in covariate means between the unmatched samples indicate that it was especially the size of non-privatized hospitals (in terms of beds) that was likely to be larger than that of privatized hospitals. After propensity score matching, the differences in covariate means between the privatized and non-privatized hospitals were substantially smaller than those seen in the pre-matching distribution. All of the differences between the groups were less than 6% in the post-matching distribution. Table 6 summarizes the results of Hotelling’s T-square test. Reassuringly, the balancing conditions were satisfied within each propensity score quartile (i.e. the *P* values for all quartiles were substantially higher than 0.10).

**Table 6:** Results from Hotelling’s T square test by propensity score quartile

Quartile	T squared statistics	*F* test statistics	*P* values
FIRST	3.593	0.551	0.768
SECOND	4.647	0.712	0.641
THIRD	4.066	0.615	0.717
FOURTH	4.933	0.746	0.615

The regression results for our first model, which used DEA efficiency scores as dependent variable in the regressions, are summarized in Table 7 and stratified according to whether public hospitals were converted to private for-profit or private non-profit status. Two sets of regression results are shown for each type of conversion: one based on the unmatched samples and the other based on the samples after propensity score matching. The coefficients shown in Table 7 are those for the difference-in-difference interaction between the variables PRIV and POST and can be interpreted as marginal effects. The interaction terms identify the changes in efficiency after a hospital was privatized relative to changes in efficiency in the comparator group.

**Table 7:** Regression results for the efficiency model

Post-privatization year	Public->For-profit		Public->Non-profit	
	Unmatched	Matched	Unmatched	Matched
1st year after privatization	0.005 (0.008)	0.021** (0.011)	0.029** (0.012)	0.050** (0.021)
2nd year after privatization	0.019** (0.008)	0.036*** (0.011)	0.017 (0.015)	0.053** (0.024)
3rd year after privatization	0.023*** (0.009)	0.041*** (0.013)	0.020 (0.015)	0.045* (0.024)
4th year after privatization	0.029*** (0.009)	0.049*** (0.013)	0.008 (0.017)	0.037 (0.027)

*P ≤ 0.10; **P ≤ 0.05; ***P ≤ 0.01; SE in parentheses

The regression results for public hospitals that were converted to private for-profit status show that there was a significant increase in efficiency compared to the control group starting the second year after privatization in the unmatched sample and even in the first year after privatization in the matched sample. Throughout all estimations of our efficiency model, the effects of this change in status increased with the number of years after privatization. For instance, four years after their conversion to private for-profit status, the formerly public hospitals experienced an increase in efficiency that was 2.9 to 4.9% greater than that seen among non-privatized hospitals over the same period. In the matched samples, the standard errors were larger than in the pre-matched sample. This can be attributed, in part, to the smaller size of the control group; nevertheless, the general finding remained the same. The estimations also suggest that the increase in efficiency seen in public hospitals that were converted to private for-profit status is permanent and not simply a transitional phenomenon after which hospital operations revert to their pre-privatization state.

In the unmatched sample, the regression results for public hospitals that were converted to private non-profit status show that, at least in the first year after privatization, there was a significant increase in efficiency of 2.9% compared to the control group. For years 2 through 4 after privatization, we observed no significant effects apart from those seen in year 2 and 3 in the matched sample. In the matched sample, public hospitals converted to private non-profit status showed a significant increase in efficiency of 5.3% in the second year after privatization and 4.5% in the third year after privatization compared to the non-privatized hospitals. Throughout most estimations, however, the effects of a conversion to private non-profit status decreased as the number of years after privatization increased. This points to a transitory rather than a permanent effect.

**Fig. 1:**
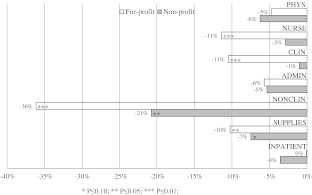
Changes in staffing (FTEs) and expenditure on supplies per case after conversion to for-profit or non-profit status compared to the control group. Note: Changes in staffing and supplies per case-mix-adjusted inpatient hospital case four years after conversion to for-profit or non-profit status compared to the control group of non-privatized hospitals. *FTE* full-time equivalents

To gain a better understanding of these findings and the restructuring that occurred after hospitals were converted to private for-profit and non-profit status in our sample, we conducted a series of exploratory regressions. Using the same model specification to identify the effect of privatization on the quantity and type of resource use (i.e. employing input variables from the first-stage analysis as dependent variables of the second-stage regression) compared to the non-privatized public hospitals, we found that hospitals that were converted to private for-profit status substantially reduced all of the analyzed labor inputs during the post-privatization period with the exception of physicians and administrative staff (i.e. nursing staff, other clinical staff and other nonclinical staff). This finding can potentially be explained by the two lines of internal authority—medical and managerial—that characterize most hospitals: In contrast to nurses and technical staff, physicians are often part of the top management and make decisions on the allocation of resources. Being clinicians or former clinicians themselves, members of top management may be predisposed to avoiding reductions in the number of physicians. Another explanation may be that avoiding physician lay-offs is a strategy to reduce resistance to change by compensating physicians for coping with the organizational adjustments associated with privatization. For hospitals converted to non-profit status reductions of labor input tend to be smaller compared to conversions to for-profit status. Figure 1 shows the changes in staffing and supplies per case-mix-adjusted inpatient hospital case that can be seen four years after the conversion to private for-profit or non-profit status compared to the control group of non-privatized hospitals. We use the fixed effects specification based on the matched sample for these illustrations.

To explore our results yet further, we examined the interaction effects between a given privatization (difference-in-difference coefficient) and the competitive environment (HHI) in which this privatization took place. In their summary of the theoretical literature, Sheshinski and López-Calva [65] argue that privatized organizations are likely to have lower efficiency gains in non-competitive markets than they would under the discipline of a competitive market. They assume that organizations in monopolistic or oligopolistic markets restrict output and have higher average costs and lower efficiency than competitive organizations. Rosko [58–60] and Rosko and Chilingerian [61], however, have found that efficiency in the US hospital sector is negatively related to market competition. Using HHI as a proxy for competition in our present study, we were unable to observe any effect of this variable on efficiency after privatization.

We also examined the interaction effects between privatization and the introduction of yardstick competition through DRGs in the year 2004 in Germany. There is reason to believe that efficiency gains are larger for privatizations after the introduction of yardstick competition, especially in the case of hospitals converted to private for-profit status [63, 66–68]. We used a dummy variable to model the pre-DRG and the DRG period. In doing so, we defined each of the years 2001 through 2004 as the cut-off point between pre-DRG and DRG periods because hospitals may have changed their behavior after the introduction of DRGs or beforehand based on their knowledge of the new system and its upcoming implementation. Table 8 summarizes the corresponding regression results based on a fixed effects regression model.

**Table 8:** Changes in efficiency after privatization in the DRG era compared to the pre-DRG era

Post-privatization period	Matched sample			
	t + 1	t + 2	t + 3	t + 4
Public->For-profit				
2001 cut-off point	−0.020 (0.016)	−0.019 (0.018)	−0.051*** (0.020)	0.012 (0.028)
2002 cut-off point	−0.021 (0.014)	−0.012 (0.018)	−0.037** (0.018)	−0.035 (0.022)
2003 cut-off point	−0.018 (0.014)	−0.032** (0.015)	−0.037** (0.018)	−0.039** (0.019)
2004 cut-off point	−0.026* (0.014)	−0.023* (0.015)	−0.047*** (0.016)	−0.042** (0.018)
Public->Non-profit				
2001 cut-off point	−0.015 (0.031)	−0.001 (0.038)	−0.030 (0.031)	−0.035 (0.055)
2002 cut-off point	−0.015 (0.031)	−0.026 (0.037)	−0.030 (0.033)	−0.057* (0.035)
2003 cut-off point	−0.015 (0.031)	−0.026 (0.037)	−0.050 (0.033)	−0.057* (0.035)
2004 cut-off point	−0.003 (0.033)	−0.049 (0.036)	−0.050 (0.033)	−0.076** (0.035)

*P ≤ 0.10; **P ≤ 0.05; ***P ≤ 0.01; SE in parentheses

t − 1 is defined as the year before privatization occurred, while t + 1, t + 2, t + 3 and t + 4 represent the four post-privatization years.

The regression results for hospitals that were converted to private for-profit status reveal a significant negative association between post-privatization performance and the introduction of DRGs. In particular, the efficiency gains seen in hospitals converted to private for-profit status were substantially lower in the DRG era compared to those seen in the control group. The introduction of DRGs induced yardstick competition in the German hospital market, setting strong incentives to increase efficiency and stimulating restructuring efforts across all types of ownership. Our findings thus suggest that it became more challenging for hospitals that were converted to private for-private status in the DRG era to realize further efficiency gains. In the model estimated for hospitals that were converted to private non-profit status, the interaction term between post-privatization performance and the introduction of DRGs was not significant.

The results of our regression analyses for for-profit and non-profit privatization indicate that the efficiency effects of privatization depend on the type of privatization. There are several potential reasons for-profit hospitals generating permanent efficiency gains after privatization while non-profit hospitals do not. Our results show that public hospitals that were converted to private for-profit status realized their substantial efficiency gains by markedly reducing all labor inputs (with the exception of physicians and administrative staff) and expenditure on supplies. In contrast, non-profit privatization only had a significant negative effect on the non-medical categories of personnel and expenditure on supplies. In short, the employment-reducing effect of for-profit privatization, especially in terms of the medical categories of staff (i.e. nursing staff and other clinical staff) was larger than that of non-profit privatization. These findings are similar to those of Picone et al. [26], and Shen [28]. Regarding the incentive structures of private non-profit providers, agency theory and property-rights theory predict that the owners and managers of non-profit organizations frequently diverge from cost- or inefficiency-minimizing behavior and instead maximize quality, quantity, and/or prestige [69, 70]. In other words, private non-profit hospitals may avoid reductions in staff with the intention to avoid reductions in the quality of care. The positive relationship between the staff-to-patient ratio and quality of care has been confirmed previously by a number of studies (e.g., Aiken et al. [71, 72]). Therefore, other measures not considered in our analyses, such as patient satisfaction or other measures of quality of care, might have led to different results.

In line with the evidence found in studies from other countries, especially the U.S., the evidence from Germany suggests that private for-profit ownership is associated with a strong focus on profit-maximization [20, 73, 74]. According to agency theory, the owners of private for-profit hospitals may use profits as their measure of a manager’s success and can limit divergences from their interest by making the manager’s compensation a positive function of these profits and are thus more likely to achieve greater efficiency [69, 75–77]. The income of physicians in private for-profit hospitals can also be tied to a hospital’s financial performance. Within public and private non-profit hospitals, the income of individual decision makers is rarely tied to a hospital’s performance, creating little incentive to enforce strong efficient behavior.

Further it should be considered that in the pre-DRG era private for-profit hospitals tended to focus on revenues in order to earn profits and were able to generate significantly higher revenues per case than hospitals with other forms of ownership [73, 74]. As the development of the German DRG-system progresses options to focus on revenues substantially decreased over the last years and thus especially private for-profit hospitals are very likely to increase their focus on efficiency as a kind of compensation. However, the generated profits allowed private for-profit hospitals to substantially invest in their hospital infrastructure that also might have positively affected the efficiency of their service production processes. In addition, private for-profit hospitals started earlier than private non-profit hospitals to specialize on certain diagnoses or procedures in order to realize economies of scale. Finally, these hospitals also tend to form chains and networks with other hospitals realizing better capabilities to negotiate with sickness funds and exploiting economies of scale for example by transferring knowledge or centralizing the purchase of supplies [78].

We tested the robustness of our findings in several ways. First, we employed the average in-hospital mortality rate as an additional output to adjust for variations in the quality of care between hospitals [41]. The results of our quality-adjusted efficiency model show that employing in-hospital mortality as an additional output was not associated with smaller efficiency gains (see Table 9 for the regression results of the quality-adjusted efficiency model). In fact, the regression results for hospitals that were converted to private for-profit status showed that there was even a small increase in coefficients compared to the estimations of the efficiency model. Our findings therefore do not provide any evidence that increases in technical efficiency come at the expense of the quality of care. This may be due to a decrease over the past decade in the asymmetry of information about the quality of care in the German hospital sector following several health care reforms that have aimed to improve quality assurance, such as the introduction of mandatory quality reports. Furthermore, it is important to consider that privatized hospitals are often located in very competitive regions with substantial overcapacities, which may increase the importance of quality of care as a parameter of competition. Indeed, there is evidence that private for-profit hospitals have improved their quality management and hospital outcomes precisely in order to attract patients in such settings [79].

**Table 9:** Regression results for the quality-adjusted efficiency model

	Public->For-profit		Public->Non-profit	
Post-privatization year	Unmatched	Matched	Unmatched	Matched
1st year after privatization	0.004 (0.008)	0.024** (0.011)	0.028** (0.012)	0.049** (0.021)
2nd year after privatization	0.022*** (0.008)	0.037*** (0.011)	0.015 (0.015)	0.050** (0.024)
3rd year after privatization	0.025*** (0.009)	0.043*** (0.013)	0.019 (0.015)	0.042* (0.024)
4th year after privatization	0.031*** (0.009)	0.055*** (0.013)	0.002 (0.017)	0.022 (0.027)

*P ≤ 0.10; **P ≤ 0.05; ***P ≤ 0.01; SE in parentheses

Second, we re-estimated all second-stage regressions by incorporating firm fixed effects as an additional dimension. To do so, we estimated time and firm fixed effects as a multi-way clustered regression [80]. This resulted in only minor changes in the difference-in-difference coefficients, and our findings remained robust throughout the models. Third, in other economic sectors, Parker and Martin [81] found that pre-privatization gains in efficiency can be even larger than post-privatization gains. Based on this finding, there is reason to assume that local governments make efforts to increase the efficiency of public hospitals prior to privatization to generate higher sales revenue, reducing potential gains in efficiency after privatization. In order to take account of this possibility, we re-ran our models, allowing an alternative pre-privatization period of two years. The direction of the difference-in-difference coefficients did not change throughout the models, and standard errors increased only slightly due to the reduced sample size. Bias due to pre-privatization efficiency gains was thus unlikely. Fourth we re-estimated our DEA models after eliminating those inputs (physicians and other clinical staff) having a strong correlation (r > 0.90) to other inputs used in the full DEA model. Thus, we performed 3 alternative DEA models: 1) without physicians, 2) without other clinical staff, 3) without physicians and other clinical staff. All other steps remained unchained. In the re-estimated regression models the coefficients and standard errors hardly changed compared to the full models. The only change occurred in model 3) where standard errors were smaller for non-profit privatization in the 3rd year after privatization. This likely reason for this effect is that variation of both these inputs across hospitals is larger than for most other inputs. Fifth, one could argue that our multi-step procedure to generate results may move the sample too far away from reality. Therefore, we added a further sensitivity analysis to show that results are robust even if we drastically reduce the numbers of steps performed in our analysis. In doing so, we estimated a model with the following specifications: We re-estimated the DEA model without risk-adjusted cases as output. 2) We proceeded without bootstrapping. 3) We estimated the regression based on the unmatched samples i.e. eliminated the matching step. We still see large and significant efficiency gains after for-profit privatizations, coefficients are slightly lower and standard errors tend to be larger. Altogether our study results are very robust to model changes.

Our study has a number of strengths compared to previous investigations of hospital ownership conversions. First, to our knowledge, it is the first quantitative study to examine the effects of privatization on technical efficiency using a panel data approach based on bootstrapped DEA efficiency scores. Second, our study employs a difference-in-difference matching approach to address problems arising both from causal inference and time-invariant differences. Third, our sample of privatized hospitals is large (*n* = 132), providing greater statistical power and more robust estimates than the analyses conducted in previous studies. Finally, our sample is rich, consisting of a large set of environmental and organizational characteristics. This is likely to have yielded more consistent results because it allowed us to control appropriately for determinants of efficiency other than the privatization event.

Our study also has several important limitations. First, additional inputs (e.g., capital) and additional outputs (e.g., hospital outpatient cases) would have improved our model of the hospital production process. Considering the number of hospital outpatient cases in addition to inpatient cases is often recommended in order to measure patient care output [38]. Although we had intended to include a proxy for hospital outpatient activities in our analysis (e.g., outpatient surgery), data inconsistencies and measurement errors prevented us from doing so. Second, including explanatory factors in addition to environmental and organizational characteristics might have provided a better explanation of variation in our estimates, thus potentially affecting our interpretation of the relationship between privatization and organizational efficiency. Third, our study relies on in-hospital mortality data and does not take post-hospital mortality into account, which clearly would have been preferable. This being said, Rosenthal et al. [82] found that (a) in-hospital mortality data were not biased by discharge practices and (b) using in-hospital mortality as a measure of a hospital’s quality of care leads to results similar to those obtained with 30 days post-hospital mortality. It should also be considered that mortality represents only one dimension of quality. There are numerous other ways of measuring quality of care, e.g., adherence to guidelines, waiting time, patient satisfaction, which would be worth including in a more comprehensive quality-adjusted DEA model. However, by using the above mentioned data set we are constrained to mortality as a proxy for quality. Thus, our quality-adjusted DEA model is not able to provide a comprehensive picture of the overall quality of care hospitals provide and one should take these limitations into consideration when drawing conclusions based on quality-adjusted efficiency. Moreover, one should generally be careful when interpreting quality-adjusted efficiency in a way that patients may find it acceptable to receive a lower quality of care if efficiency is high [83]. In this study the quality-adjusted efficiency model is only meant to serve as robustness check to show that efficiency gains after privatization do not change completely if mortality as one dimension of quality of care is considered in our models. Fourth, for sensitivity purposes it would have been preferable to use SFA in addition to DEA. When comparing DEA to SFA, however, Linna [84] and Webster et al. [85] found that both methods yielded comparable results when measuring hospital efficiency. Fifth, in DEA model II, we used 1 minus the in-hospital mortality rate per year, which represents an index variable. Syrjänen [39] found that mixing index and volume measures in DEA may lead to biased results for the most commonly used constant returns to scale variant of the Banker and Moorey (BM) model and Charnes, Cooper, and Rhodes (CCR) model. This problem does not apply in our context, however, because we used a variable returns to scale variant of the BCC model [39, 86]. Finally, although we improve over other studies by considering a measure for differences in case-mix among hospitals there are reasons to believe that certain potentially important differences are not captured by this measure. For instance, multimorbidity of patients and the resource use associated with this are often not adequately captured by case-mix measures such as the Elixhauser Index.

## Conclusions

In the present study, we investigated the post-acquisition effects of privatization on hospital efficiency in Germany. Our findings show that hospitals converted to private for-profit status showed an increase in efficiency after privatization that was significantly higher than that realized by their non-privatized counterparts over the same period. We defined four alternative post-privatization periods (i.e. first, second, third, and fourth year) and found that the increase in efficiency after conversion to private for-profit status did not appear to be transitory. Our results show, that hospitals that were converted to private for-profit status realized their substantial efficiency gains by markedly reducing all labor inputs (with the exception of physicians and administrative staff) and expenditure on supplies. The results of our study, which remained robust when considering in-hospital mortality as an additional output, do not support frequently voiced concerns that efficiency gains after conversion to private for-profit status are realized at the expense of quality of care. Taking the effect of the introduction of DRG payments into account, it is striking that the efficiency gains of hospitals converted to private for-profit status were significantly lower during the DRG era. We also observed an increase in efficiency one year after hospitals were converted to private non-profit status, but our estimations suggest that this effect is transitory and that hospitals converted to private for-profit status follow a different restructuring strategy than that pursued by their private non-profit counterparts.

Our results indicate that converting hospitals to private for-profit status may be an effective way to ensure better allocation of resources in the hospital sector. Before drawing policy implications, however, a number of issues must be considered. In some cases, public hospitals converted to private non-profit status may not be restructured with the intention of increasing efficiency, but rather with the aim of improving the patient experience, including the quality of care. Although our results remained robust when considering in-hospital mortality for hospitals converted to private non-profit status, other measures not considered in our analysis, such as patient satisfaction, may have led to different results after the privatization event. Moreover, it should also be taken into consideration that the time needed to develop new quality assurance systems in privatized hospitals may be longer than the period we considered in our study. Additional research is needed to investigate the long-term effects of privatization and to examine the decisions behind the approaches taken by private for-profit and private non-profit organizations in their restructuring of public hospitals.

Given the increasing importance of privatization in health systems in Germany and beyond, further studies are needed to investigate the effects of privatization on efficiency and different dimensions of care quality. Outside the hospital sector, privatization activity is also increasing in laboratory diagnostics, nursing care and rehabilitative services. The approach used in our study may be useful in investigating privatization in these other areas of care. In addition, future research should go beyond the standard administrative hospital data base to further explain the wide variation in hospital efficiency after privatization which may be due to hospital decision making, market spillover effects or other reasons.
